# Features, Behavioral Change Techniques, and Quality of the Most Popular Mobile Apps to Measure Physical Activity: Systematic Search in App Stores

**DOI:** 10.2196/11281

**Published:** 2018-10-26

**Authors:** Patrícia Simões, Anabela G Silva, João Amaral, Alexandra Queirós, Nelson P Rocha, Mário Rodrigues

**Affiliations:** 1 School of Health Sciences University of Aveiro Aveiro Portugal; 2 Center for Health Technology and Services Research University of Aveiro Aveiro Portugal; 3 School of Technology and Management at Águeda University of Aveiro Aveiro Portugal; 4 Institute of Electronics and Telematics Engineering of Aveiro University of Aveiro Águeda Portugal; 5 Department of Medical Sciences University of Aveiro Aveiro Portugal

**Keywords:** behavioral change techniques, mobile phone app, physical activity, quality, technical features, mobile phone

## Abstract

**Background:**

It is estimated that 23% of adults and 55% of older adults do not meet the recommended levels of physical activity. Thus, improving the levels of physical activity is of paramount importance, but it requires the use of low-cost resources that facilitate universal access without depleting the health system. The high number of apps available constitutes an opportunity, but it also makes it quite difficult for the layperson to select the most appropriate app. Furthermore, the information available in the app stores is often insufficient, lacks quality, and is not evidence based, and the systematic reviews fail to assess app quality using standardized and validated instruments.

**Objective:**

The objective of this study was to systematically assess the features, content, and quality of the most popular apps that can be used to measure and, potentially, promote physical activity.

**Methods:**

Systematic searches were conducted on Apple App Store, Google Play, and Windows Phone Store between December 2017 and January 2018. Apps were included if their primary objective was to assess the aspects of physical activity, if they had a user rating of at least 4, if their number of ratings was ≥100, and if they were free. Apps meeting these criteria were independently assessed by two reviewers regarding their general and technical information, aspects of physical activity, presence of behavioral change techniques, and quality. Data were analyzed using means and SDs or frequencies and percentages.

**Results:**

Of 51 apps included, none specified the age of the target group and only one mentioned the involvement of health professionals. Most apps offered the possibility to work in background (n=50) and allowed data sharing (n=40). Regarding physical activity, most apps measured steps and distance (n=11) or steps, distance, and time (n=17). Only 18 apps, all of which measured number of steps, followed the guidelines on recommendations for physical activity. On average, 5.5 (SD 1.8) behavioral change techniques were identified per app; the most frequently used techniques were “provide feedback on performance” (n=50) and “prompt self-monitoring of behavior” (n=50). The overall quality score was 3.88 (SD 0.34).

**Conclusions:**

Although the overall quality of the apps was moderate, the quality of their content, particularly the use of international guidelines on physical activity, should be improved. Additionally, a more in-depth assessment of apps should be performed before releasing them for public use, particularly regarding their reliability and validity.

## Introduction

Physical inactivity is considered as the biggest public health problem of the 21st century [1]. It is the fourth leading risk factor for global mortality, contributing to 6% of deaths globally, and is one of the main risk factors for many major noncommunicable diseases such as cardiovascular disease, diabetes, and cancer [2]. The World Health Organization (WHO) has defined the levels of physical activity per age group with an impact on health. For example, WHO recommends that adults perform at least 150 minutes of moderate-intensity or 75 minutes of vigorous-intensity aerobic physical activity every week [2]. However, 23% of adults and 55% of older adults are not meeting these recommended physical activity levels and are, thus, insufficiently active [3]. Thus, while improving the levels of physical activity is of paramount importance, it requires the use of low-cost resources that facilitate universal access without depleting the health system.

Smartphones and mobile apps constitute a potential means to promote physical activity and one that is available to potentially everyone at low or no cost. Smartphone ownership is expected to grow from 1.86 billion in 2015 to around 2.87 billion in 2020 [4]. Similarly, the number of apps, particularly those related to health and fitness, has been increasing. As of June 2018, there were 3,509,819 and 3,249,721 apps in the Google Play and Apple App Store [5,6], respectively; of them, 102,962 and 97,844 were categorized as Health and Fitness apps, respectively [7,8]. This high number of apps available constitutes an opportunity, but it also makes it quite difficult for the layperson to select the most appropriate app. Furthermore, as the information available in the app stores is often insufficient, lacks quality, and is not evidence based [9], it is essential to use standardized instruments to assess the content and quality of the available apps that claim to measure or promote physical activity in terms of what they measure and how, whether they follow international health and fitness guidelines, and their use of behavioral change techniques. Existing systematic reviews assessing apps that measure or promote physical activity have focused mainly on characterizing their content, particularly regarding the use of behavioral change techniques [10-13] because these interventions are associated with greater effectiveness [14]. Nevertheless, app quality has not usually been assessed using standardized and validated instruments. Therefore, the present study aims to systematically assess the features, content, and quality of the most popular apps that can be used to measure and, potentially, promote physical activity and that are available in the Apple App Store, Google Play, and Windows Phone Store.

## Methods

### Search Strategy

Systematic searches were conducted independently by two researchers (PS and JA) in the Apple App Store, Google Play, and the Windows Phone Store between December 2017 and January 2018. Apps were identified using the following search terms: “physical activity,” “tracker,” “distance,” and “pedometer.” The search terms were entered in the platforms separately or in combinations based on the Boolean logic.

### Inclusion Criteria and Selection Process

The selection process was conducted in two phases. In the first phase, apps were retrieved and registered in a database if they (1) were written in English or Portuguese (description and application); (2) had “physical activity” or related words featured in the keywords or text description; and (3) had a primary objective to assess the aspects of physical activity. For the purpose of this study, physical activity was defined as any movement of the body produced by the skeletal muscles that results in energy expenditure that can be objectively characterized by measuring body displacement [15,16]. Apps could be used alone or in combination with an external device (eg, physical activity tracker) or a back-office system, for example, to communicate with a health professional. Apps were excluded if they were intended for use in a clinical context only (eg, hospital or other health care context) or primarily targeted health behaviors other than physical activity (eg, diet). Two reviewers (PS and JA) independently assessed the names and descriptions of mobile apps against the inclusion criteria. Disagreements were resolved through discussion with a third reviewer (AGS). The apps identified in this first phase were then included in the second phase of selection. In this phase, a second set of criteria was used to identify the apps that would enter this review. Apps were included if (1) they had a user rating of at least 4 (scale range: 1-5) in line with previous app reviews [9,17]; (2) they had a number of ratings ≥100; and (3) they were free (as we believed these to be the most commonly used by the general population). This selection was performed between the January 25 and 30, 2018, by one reviewer (PS). Apps meeting these criteria were then installed into appropriate devices and independently assessed by two reviewers (PS and JA) using standardized forms. Disagreements were resolved through discussion with a third reviewer (AGS). When the same app was available from more than one store, it was downloaded from one store only (usually the Google Play).

### Data Extraction

Data extraction was performed using customized forms specifically designed for this assessment and that had been piloted using 3 apps (“C25K 5K Trainer,” “Wokamon–Fitness Game,” and “Pedometer, Step Counter & Weight Loss Tracker”) to standardize the rationale and procedures. Data retrieved from the platforms and apps covered the following:

1. The general and technical information, which was assessed using both the app classification subscale from the Mobile Applications Rating Scale (MARS) [18] and a set of criteria defined by the authors of the present study (involvement of health professionals in the development of the app, presence or absence of a back-office, possibility to connect to other peripheral devices, possibility to work in the background, calendarization, possibility to give geographic information, types of authorizations needed, and the existence of videos showing exercises or other information), totalizing a maximum of 12 features.
2. The aspects of physical activity such as the number of steps, distance, time, and velocity; whether they could be used indoors or outdoors; and the accuracy of the content in accordance with guidelines, such as the WHO recommendations of at least 150 minutes of moderate-intensity or 75 minutes of vigorous-intensity aerobic physical activity in a week [2] and the recommendation of 10,000 steps per day [19].
3. The presence or absence of behavioral change techniques, which were assessed using the taxonomy of behavioral change techniques developed by Abraham & Michie [20]; this taxonomy includes 26 behavioral change techniques, but 3 of them (provide information on consequences, provide general encouragement, and provide information about other’s approval) showed low interrater reliability [20]. Thus, they were not included, and the remaining 23 behavioral change techniques were categorized as being present or absent.

### Quality Assessment

App quality was assessed using the App Quality Ratings subscale of MARS, which includes 19 items grouped into 4 sections: (1) engagement (entertainment, interest, customization, interactivity, target group); (2) functionality (performance, ease of use, navigation, gestural design); (3) aesthetics (layout, graphics, visual appeal); and (4) information quality (accuracy of app description, goals, quality and quantity of information, visual information, credibility, evidence base) [18]. All items were assessed using a 5-point scale (1: inadequate to 5: excellent). A mean score for each of the 4 subscales as well as an overall score resulting from the mean of the 4 subscale scores was calculated [18]. MARS has been found to be reliable and to have very high to excellent internal consistency [18]. App quality assessment was conducted independently by two reviewers (PS and JA). Disagreements between the two reviewers were resolved through discussion with a third reviewer (AGS).

### Statistical Analysis

Data were analyzed using means and SDs for continuous variables and frequencies and percentages for nominal and ordinal variables. The agreement between the reviewers was characterized using agreement percentages for nominal variables (eg, behavioral change techniques) and the intraclass correlation coefficient (ICC; model: two-way mixed effects, absolute agreement) for continuous variables (eg, MARS subscales and total score). The acceptable level of percentage agreement and interrater reliability was set at 80% and 0.70, respectively [21].

## Results

### App Selection

**Figure 1:**
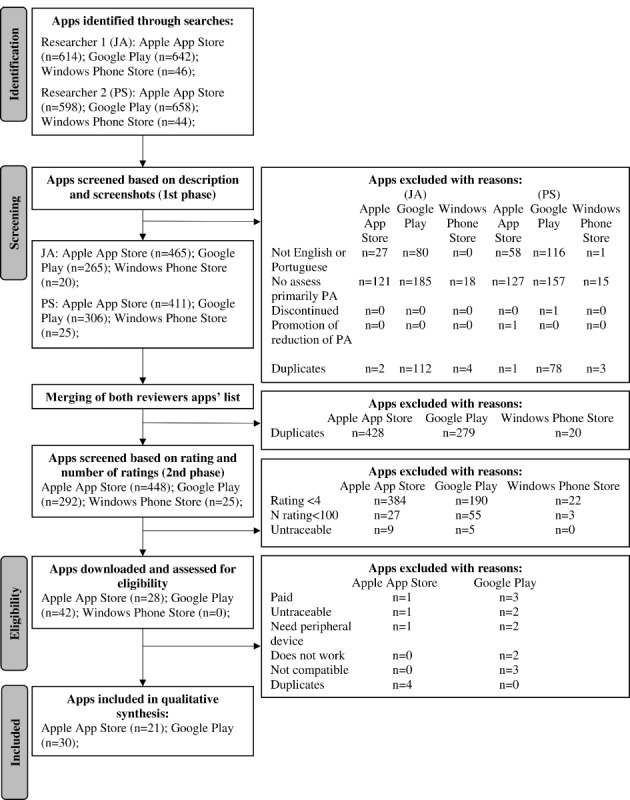
Flowchart of the selection process for apps included in the study. PA: physical activity.

A flowchart for the app selection process is presented in Figure 1. One reviewer (JA) identified 614 apps in the Apple App Store, 642 apps in the Google Play, and 46 apps in the Windows Phone Store, while the other reviewer (PS) identified 598 apps in the Apple App Store, 658 apps in the Google Play, and 44 apps in the Windows Phone Store. After screening for eligibility against the inclusion and exclusion criteria in both phases 1 and 2, 51 apps were selected for this assessment (21 apps from the Apple App Store and 30 apps from the Google Play; Multimedia Appendix 1).

### General Characteristics of the Selected Apps

The general characteristics of the included apps are presented in Table 1. Of the 51 free apps, 27 had an upgraded version with an average cost of 3.27€ (range: 0.89€-10.99€); the mean user rating was 4.39 (SD 0.25), and the mean number of user ratings was 27,852.96 (SD 12,339.92). None of the apps specified the age of the target group, but 3 apps were not recommended for children and adolescents (age<18 years). Only 1 app (“The Walk: Fitness Tracker Game” from the Apple App Store, which was developed by the National Health Service and Department of Health in the United Kingdom) of the 51 included in this study mentioned the involvement of health professionals in its development. The mean number of app features per app was 3.2 (SD 1.7) out of a maximum of 12 (range: 1-7). Most apps offered the possibility to work in the background (50/51, 98%), most allowed data sharing (40/51, 78%), and none required internet access to measure the physical activity. In terms of the physical activity types, most apps measured steps and distance (11/51, 22%) or steps, distance, and time (17/51, 33%); 96% (49/51) apps could be used both outdoors and indoors, and the remaining 2 apps (“Sports Tracker for All Sports” and “Walking Odometer Pro-GPS Pedometer & Fitness”) were for outdoor use only as they needed a global positioning system signal. Only 18 apps, all of which measured the number of steps, followed the guidelines recommended for physical activity (number of steps).

### Presence of Behavioral Change Techniques

**Figure 2:**
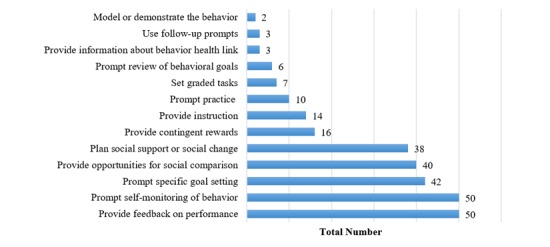
Frequencies of the 23 behavioral change techniques presented in the included apps (N=51).

The percentage of agreement between reviewers regarding the presence of behavioral change techniques in the included apps was 93.1%. The mean number of behavioral change techniques per app was 5.5 (SD 1.8), and overall, all apps included at least 3 behavioral change techniques. The maximum number of behavioral change techniques was 10 in one app (“Pedometer-Six pack Workout” from the Google Play). Commonly included behavioral change techniques were “Provide feedback on performance” (50/51, 98%), “Prompt self-monitoring of behavior” (50/51, 98%), “Prompt specific goal setting” (42/51, 82%), “Provide opportunities for social comparison” (40/51, 78%), and “Plan social support or social change” (38/51, 75%). Some behavioral change techniques were not found in any of the included apps: “Prompt intention formation,” “Prompt barrier identification,” “Teach to use prompts or cues,” “Agree on behavioral contract,” “Prompt identification as a role model,” “Prompt self-talk,” “Relapse prevention,” “Stress management,” “Motivational interviewing,” and “Time management.” Figure 2 shows the absolute frequencies for the behavioral change techniques used in the 51 apps included in the present assessment.

**Table 1:** General characteristics of the included apps (N=51).

Characteristics		Value
**Store, n (%)**		
	Apple App Store	21 (41)
	Google Play	30 (59)
**User rating, mean (SD)**		
	Average rating	4.4 (0.3)
	Average number of user ratings	27,853 (12,339.9)
Health professional involvement, n (%)		1 (2)
**Age (years), n (%)**		
	All age groups	48 (94)
	Adults only (age>17)	2 (4)
	Adults and adolescents (age>12)	1 (2)
**Number of app features (out of a maximum of 12), mean (SD)**		3.2 (1.7)
	Allows sharing, n (%)	40 (78)
	Allows password protection, n (%)	2 (4)
	App community, n (%)	17 (33)
	Calendarization, n (%)	4 (8)
	Connects with peripheral devices, n (%)	9 (18)
	Geographic information, n (%)	10 (20)
	Has a back-office, n (%)	7 (14)
	Has videos showing exercises or other information, n (%)	4 (8)
	Needs internet to work, n (%)	0 (0)
	Requires log-in, n (%)	7 (14)
	Sends reminders, n (%)	11 (22)
	Works in background, n (%)	50 (98)
**Type of measurements for physical activity (PA), n (%)**		
	Steps only	2 (4)
	Time only	2 (4)
	Steps and distance	11 (22)
	Distance and time	1 (2)
	Steps, distance, and time	17 (33)
	Steps, distance, and velocity	3 (6)
	Distance, time, and velocity	6 (12)
	Steps, distance, time, and velocity	9 (18)
**Guidelines for PA, n (%)**		
	Follows guidelines^a^	18 (35)
	Does not follow guidelines	33 (65)
**Environment where apps measure PA, n (%)**		
	Indoors and outdoor	49 (96)
	Outdoors only	2 (4)

^a^All apps that followed the guidelines measured the number of steps.

### App Quality

Overall, the reliability regarding the assessment of the app quality was excellent with an ICC of 0.93 (95% CI 0.70-0.97) for the MARS total score. For subscale A, the reliability for the MARS ICC was 0.83 (95% CI 0.63-0.92); for subscale B, it was 0.88 (95% CI 0.78-0.93); for subscale C, it was 0.90 (95% CI 0.78-0.95); and for subscale D, it was 0.93 (95% CI 0.70-0.97). The mean MARS total score was 3.88 (SD 0.34) out of 5; it ranged between 3.16 (“StepWalk Pedometer” from the Google Play) and 4.41 (“Accupedo + Pedometer” also from the Google Play). The subscale with the highest score was “Functionality” (mean 4.30 [SD 0.68]), followed by “Aesthetics” (mean 4.15 [SD 0.68]), “Information Quality” (mean 3.78 [SD 0.28]), and “Engagement” (mean 3.28 [SD 0.34]).

## Discussion

### Main Findings and Comparison With Prior Work

This study assessed the features, content, and quality of the most popular physical activity apps available in the Apple App Store, Google Play, and Windows Phone Store. Most of the included apps targeted all age groups and none specifically targeted children, adolescents, or older adults. Schoeppe et al [17] conducted a similar systematic assessment of apps that targeted diet, physical activity, and sedentary behavior in children and adolescents and found only 18 apps specifically designed for children and adolescents that targeted physical activity. Nevertheless, WHO recommendations for physical activity for children, adolescents, adults, and older adults are different [2]. Moreover, it is likely that an app that is adequate for and captivates adolescents would be different from an app that is adequate and easy to use by older adults, who are also a high-risk age group for low levels of physical activity [3]. When assessing whether the apps considered the established guidelines for physical activity, 18 of the 42 apps that measured the number of steps followed the guidelines (ie, mentioned 10,000 steps per day); however, none followed the WHO guidelines for intensity, duration, and frequency (ie, at least 150 minutes of moderate-intensity or 75 minutes of vigorous-intensity aerobic physical activity per week). The lack of apps following the WHO recommendations was also mentioned in the review of Knight et al [22], highlighting the need to develop apps that measure intensity, frequency, and duration and that make recommendations based on the established guidelines. For example, an app could register the weekly frequency and duration of physical activity and match these against the guidelines every week. The app could also prompt the user to classify the intensity of the activity based on existing scales such as the Borg scale [23].

Of the 51 reviewed apps, only 1 (“The Walk: Fitness Tracker Game” from the Apple App Store) mentioned the involvement of health professionals in its development. The low involvement of health professionals in the development of apps has also been found in other reviews of physical activity apps [24], pain apps [25], obesity apps [26], and apps that target medication adherence [27]. The involvement of health professionals in the development of apps targeting health behaviors is crucial if the app content is to be evidence based, ie, based on information that is scientifically accepted and appropriate for the target population, and it also contributes to decrease the possibility of apps being harmful and misleading.

Most apps included technical features such as the possibility to share the accomplishments on social media, such as Facebook, and the possibility to work in the background. The possibility of sharing accomplishments on social media can help individuals stay motivated and increase the levels of physical activity; however, there are also concerns regarding what to share and with whom [28].

The number of behavioral change techniques included in the apps ranged between 3 and 10 with a mean of 6 per app. This is consistent with previous reviews that found the mean number of behavioral change techniques per app to range between 4 and 8 [10-13,24]. Nevertheless, the optimal number of behavioral change techniques per app remains unclear. According to Michie et al [14], interventions targeting diet and physical activity that include feedback on performance combined with self-monitoring, goal setting, intention formation, and review of goals are associated with greater effectiveness. While providing feedback on performance, goal setting, and self-monitoring were among the most common behavioral strategies present in the reviewed apps, intention formation and goal review were not.

The quality of the reviewed apps was moderate, with a mean MARS total score of 3.88. Similar to other reviews that assessed app quality using MARS [9,17], the domains with the highest scores were functionality and aesthetics, while the domains with the lowest scores were engagement and information quality. These results suggest that developers are more concerned with the ease of use, functionality, and aesthetics than with engagement and content based on high-quality evidence. Furthermore, none of the reviewed apps had been assessed for usability, validity, reliability, and effectiveness, which raises further concerns regarding the quality and impact of the apps that are freely available to everyone in the commonly used app stores. These aspects should be assessed before the apps are released, or at least, a reference to their absence should be made.

### Limitations

In this study, only the most popular apps were assessed, which limits the generalizability of results to all available apps. Another limitation was the short period used for the individual assessment of each app, which may have led to the nondetection of some features (for example, presence of reminders) and behavioral change techniques that required more time, such as “Use of follow-up prompts” and “Prompt review of behavioral goals,” and prevented the assessment of engagement to the apps. However, app quality was assessed by two independent reviewers using a standardized instrument, which minimized the potential errors. In addition, the apps were tested by the researchers only and not by any potential users.

### Conclusion

The results suggest that the popular apps for measuring and, potentially, promoting physical activity are of moderate quality. The app content quality, particularly the use of international guidelines on physical activity, could be improved. Furthermore, based on the findings from this assessment, we suggest that health professionals should be involved in the development of apps targeting health behaviors; that apps should be developed considering the target group and the respective recommendations for physical activity established by the WHO; and that more in-depth assessments, particularly for reliability (consistency of results) and validity (accuracy of results), be performed before apps are released to the general public, which would allow the public to make more informed choices.

## Appendix

Multimedia Appendix 1 List of the apps included and their major characteristics.

## Abbreviations

ICC: intraclass correlation coefficient
MARS: Mobile Applications Rating Scale
WHO: World Health Organization
