# Supplementary material for: Features, Behavioral Change Techniques, and Quality of the Most Popular Mobile Apps to Measure Physical Activity: Systematic Search in App Stores
Source: JMIR Mhealth Uhealth. 2018 Oct 26;6(10):e11281. doi: 10.2196/11281 (PMC6229520; doi:10.2196/11281)
Supplement: Multimedia Appendix 1 [file mhealth_v6i10e11281_app1.pdf]

Multimedia Appendix - List of the apps included in the review and their major characteristics.

Name of apps (version)	Developer Store	User rating	Target group	App features (0-12)	Type of measurements for PA	Guideline for PA	Behaviour Change Techniques (0-23)	MARS A	MARS B	MARS C	MARS D	MARS Total
**C25K® 5K Trainer (v. 4.7)**	Zen Labs LLC (CA)<br><br>Apple App Store	4.8	General	n=5;<br>Allows sharing;<br>App community;<br>Calendarization;<br>Sends reminders;<br>Works in background;	Time;	No;	n=9;<br>Provide feedback on performance;<br>Prompt self-monitoring of behaviour;<br>Prompt specific goal setting;<br>Provide opportunities for social comparison;<br>Provide contingent rewards;<br>Provide instructions;<br>Prompt practice;<br>Set graded tasks;<br>Use follow-up prompts;	3.00	4.75	4.67	4.00	4.10
**Wokamon Fitness Game (v. 3.6)**	Noodum Co. Ltd<br><br>Apple App Store	4.5	General	n=4;<br>Allows sharing;<br>App community;<br>Connects with peripheral devices; Works in background;	Steps; Distance; Time;	No;	n=5;<br>Provide feedback on performance;<br>Prompt self-monitoring of behaviour;<br>Provide opportunities for social comparison;<br>Plan social support or social change;<br>Provide contingent rewards;	3.60	4.25	4.00	4.00	3.96
**Pedometer. Step Counter & Weight Loss Tracker (v. p5.1.2)**	Pacer Health<br><br>Google Play	4.6	General	n=6;<br>Allows sharing;<br>App community;<br>Connects with peripheral devices; Has videos showing exercises and/or other information;<br>Sends reminders;<br>Works in background;	Steps; Distance; Time; Velocity;	Yes; Steps;	n=8;<br>Provide feedback on performance;<br>Prompt self-monitoring of behaviour;<br>Prompt specific goal setting;<br>Provide opportunities for social comparison;<br>Plan social support or social change;<br>Provide instructions;<br>Prompt practice;<br>Use follow-up prompts;	3.60	4.75	5.00	4.17	4.38

**10K Trainer by C25K® (v. 6.0)**	Zen Labs LLC (CA)<br><br>Apple App Store	4.7	General	n=5;<br>Allows sharing;<br>App community;<br>Calendarization;<br>Sends reminders;<br>Works in background;	Time;	No;	n=9;<br>Provide feedback on performance;<br>Prompt self-monitoring of behaviour;<br>Prompt specific goal setting;<br>Provide opportunities for social comparison;<br>Provide contingent rewards;<br>Provide instructions;<br>Prompt practice;<br>Set graded tasks;<br>Use follow-up prompts;	3.00	4.75	4.67	4.00	4.10
**Accupedo Pedometer (v. 3.6.5)**	Corusen LLC<br><br>Apple App Store	4.7	General	n=2;<br>Allows sharing;<br>Works in background;	Steps; Distance; Time; Velocity;	Yes; Steps;	n=5;<br>Provide feedback on performance;<br>Prompt self-monitoring of behaviour;<br>Prompt specific goal setting;<br>Provide opportunities for social comparison;<br>Plan social support or social change;	3.60	4.25	4.33	3.29	3.87
**ActivityTracker Pedometer (v. 2.6)**	Bits&Coffee Ltd.<br><br>Apple App Store	4.4	General	n=4;<br>Allows sharing;<br>Connects with peripheral devices; Sends reminders; Works in background;	Steps; Distance; Time;	No;	n=5;<br>Provide feedback on performance;<br>Prompt self-monitoring of behaviour;<br>Prompt specific goal setting;<br>Provide opportunities for social comparison;<br>Plan social support or social change;	3.60	4.50	4.67	4.33	4.28

**Cash for Steps (v. 1.31)**	Taras Bekhta<br><br>Apple App Store	4.5	Adults (age >17 years)	n=5;<br>Allows sharing;<br>App community;<br>Geographic information;<br>Requires login;<br>Works in background;	Steps; Distance;	No;	n=5;<br>Provide feedback on performance;<br>Prompt self-monitoring of behaviour;<br>Provide opportunities for social comparison;<br>Plan social support or social change;<br>Provide contingent rewards;	2.80	3.75	3.33	3.50	3.35
**Fit Simply - Pedometer to Watch your Steps (v. 2.2)**	Alaric Cole<br><br>Apple App Store	4	General	n=2;<br>Allows sharing;<br>Works in background;	Steps;	Yes; Steps;	n=6;<br>Provide feedback on performance;<br>Prompt self-monitoring of behaviour;<br>Prompt specific goal setting;<br>Provide opportunities for social comparison;<br>Plan social support or social change;<br>Provide contingent rewards;	3.00	4.25	4.00	3.75	3.75
**Pedometer α (v. 1.1.9)**	ITO Technologies. Inc.<br><br>Apple App Store	4.6	General	n=2;<br>Allows sharing;<br>Works in background;	Steps; Distance; Time; Velocity;	No;	n=4;<br>Provide feedback on performance;<br>Prompt self-monitoring of behaviour;<br>Provide opportunities for social comparison;<br>Plan social support or social change;	3.20	4.75	5.00	3.67	4.15
**Pedometer++ (v. 3.2.6)**	David Smith & Cross Forward Consulting. LLC<br><br>Apple App Store	4.7	General	n=1;<br>Works in background;	Steps; Distance;	Yes; Steps;	n=4;<br>Provide feedback on performance;<br>Prompt self-monitoring of behaviour;<br>Prompt specific goal setting;<br>Provide contingent rewards;	3.20	4.00	4.00	3.83	3.76

**Walking for Weight Loss (v. 4.6.1)**	Grinasys Corp<br><br>Apple App Store	4.3	General	n=4;<br>Allows sharing;<br>Calendarization;<br>Connects with peripheral devices; Works in background;	Distance; Time; Velocity;	No;	n=8;<br>Provide feedback on performance;<br>Prompt self-monitoring of behaviour;<br>Prompt specific goal setting;<br>Provide opportunities for social comparison;<br>Plan social support or social change;<br>Provide instructions;<br>Set graded tasks;<br>Provide information about behaviour health link;	3.20	4.25	4.00	4.50	3.99
**Runkeeper—GPS Running Tracker (v. 8.7)**	FitnessKeeper. Inc<br><br>Apple App Store	4.5	General	n=7;<br>Allows sharing;<br>App community;<br>Connects with peripheral devices;<br>Geographic information;<br>Requires login;<br>Sends reminders;<br>Works in background;	Distance; Time; Velocity;	No;	n=9;<br>Provide feedback on performance;<br>Prompt self-monitoring of behaviour;<br>Prompt specific goal setting;<br>Provide opportunities for social comparison;<br>Plan social support or social change;<br>Provide contingent rewards;<br>Provide instructions;<br>Prompt practice;<br>Prompt review of behavioural goals;	3.60	4.75	5.00	3.83	4.30
**Runtastic Steps: Step Counter & Pedometer (v. 2.1)**	Runtastic<br><br>Google Play	4.2	General	n=6;<br>Allows sharing;<br>App community;<br>Connects with peripheral devices; Has a back-office;<br>Requires login;<br>Works in background;	Steps; Distance; Time;	Yes; Steps;	n=7;<br>Provide feedback on performance;<br>Prompt self-monitoring of behaviour;<br>Prompt specific goal setting;<br>Provide opportunities for social comparison;<br>Plan social support or social change;<br>Provide instructions;<br>Prompt information about behaviour health link;	3.40	4.25	5.00	3.71	4.09

**Sports Tracker for All Sports (v. 4.2.2)**	Sport Tracking Technologies Ltd.<br><br>Apple App Store	4.8	General	n=7;<br>Allows sharing;<br>App community;<br>Connects with peripheral devices;<br>Geographic information; Has a back-office;<br>Requires login;<br>Works in background;	Distance; Time; Velocity;	No;	n=5;<br>Provide feedback on performance;<br>Prompt self-monitoring of behaviour;<br>Prompt specific goal setting;<br>Provide opportunities for social comparison;<br>Plan social support or social change;	3.80	5.00	5.00	3.75	4.39
**Step It Up (v.1.10.1)**	Bean's Bytes. LLC<br><br>Apple App Store	4.7	General	n=1;<br>Works in background;	Steps; Distance;	No;	n=3;<br>Provide feedback on performance;<br>Prompt self-monitoring of behaviour;<br>Prompt specific goal setting;	2.80	4.00	3.00	3.60	3.35
**Steps Pedometer & Step Counter Activity Tracker (v. 1.4.3)**	Supercritical Flow<br><br>Apple App Store	4.2	General	n=2;<br>Allows sharing;<br>Works in background;	Steps; Distance; Time;	Yes; Steps;	n=5;<br>Provide feedback on performance;<br>Prompt self-monitoring of behaviour;<br>Prompt specific goal setting;<br>Provide opportunities for social comparison;<br>Plan social support or social change;	3.20	4.75	4.00	3.50	3.86
**Steps: Pedometer step counter (v. 2.1)**	Adappt LLC<br><br>Apple App Store	4.6	General	n=3;<br>Allows sharing;<br>App community;<br>Works in background;	Steps; Distance;	Yes; Steps;	n=5;<br>Provide feedback on performance;<br>Prompt self-monitoring of behaviour;<br>Prompt specific goal setting;<br>Provide opportunities for social comparison;<br>Plan social support or social change;	3.40	4.25	4.00	3.75	3.85

**StepsApp Pedometer (v. 4.5.7)**	StepsApp<br><br>Google Play	4.7	General	n=2;<br>Allows sharing;<br>Works in background;	Steps; Distance; Time;	Yes; Steps;	n=5;<br>Provide feedback on performance;<br>Prompt self-monitoring of behaviour;<br>Prompt specific goal setting;<br>Provide opportunities for social comparison;<br>Plan social support or social change;	3.60	4.75	4.33	4.00	4.17
**StepUp Pedometer Step Tracker (v. 1.0.42)**	StepUp Lab<br><br>Google Play	4.4	General	n=6;<br>Allows sharing;<br>App community;<br>Connects with peripheral devices; Has a back-office;<br>Requires login;<br>Works in background;	Steps; Distance;	Yes; Steps;	n=6;<br>Provide feedback on performance;<br>Prompt self-monitoring of behaviour;<br>Prompt specific goal setting;<br>Provide opportunities for social comparison;<br>Plan social support or social change;<br>Prompt review of behavioural goals;	3.40	3.75	3.67	4.00	3.70
**Stepwise Pedometer (v. 3.1.22)**	Progress Concept Limited<br><br>Apple App Store	4.6	General	n=3;<br>Allows sharing;<br>Sends reminders;<br>Works in background;	Steps; Distance; Time; Velocity;	Yes; Steps;	n=6;<br>Provide feedback on performance;<br>Prompt self-monitoring of behaviour;<br>Prompt specific goal setting;<br>Provide opportunities for social comparison;<br>Plan social support or social change;<br>Prompt practice;	3.20	4.50	4.00	3.83	3.88

**Stepz - Step Counter (v. 2.3.1)**	Michael Szumielewski<br><br>Apple App Store	4.6	General	n=3;<br>Allows sharing;<br>App community;<br>Works in background;	Steps; Distance;	Yes; Steps;	n=7;<br>Provide feedback on performance;<br>Prompt self-monitoring of behaviour;<br>Prompt specific goal setting;<br>Provide opportunities for social comparison;<br>Plan social support or social change;<br>Provide contingent rewards;<br>Prompt review of behavioural goals;	3.60	4.00	4.33	4.00	3.98
**The Walk: Fitness Tracker Game (v. 2.0.3)**	Six to Start<br><br>Apple App Store	4.2	Adults and Adolescent (Age>12 years)	n=2;<br>Allows sharing;<br>Works in background;	Distance; Time;	No;	n=6;<br>Prompt specific goal setting;<br>Provide opportunities for social comparison;<br>Plan social support or social change;<br>Provide contingent rewards;<br>Provide instructions;<br>Set graded tasks;	4.00	4.50	5.00	4.00	4.38
**Track My Steps - Pedometer (v. 1.3)**	Zen Labs LLC (CA)<br><br>Apple App Store	4.6	General	n=3;<br>Allows sharing;<br>App community;<br>Works in background;	Steps; Distance; Time;	Yes; Steps;	n=5;<br>Provide feedback on performance;<br>Prompt self-monitoring of behaviour;<br>Prompt specific goal setting;<br>Provide opportunities for social comparison;<br>Plan social support or social change;	3.60	4.25	4.00	4.00	3.96
**Walker - Pedometer Lite (v. 2.5.9)**	Amuser Labo<br><br>Apple App Store	4.5	General	n=4;<br>Allows sharing;<br>Allows password-protection;<br>Geographic information;<br>Works in background;	Steps; Distance; Time;	No;	n=4;<br>Provide feedback on performance;<br>Prompt self-monitoring of behaviour;<br>Provide opportunities for social comparison;<br>Plan social support or social change;	3.00	4.50	3.67	3.33	3.63

**Walker - Pedometer M7 - M11 (v. 2.4.13)**	Amuser Labo<br><br>Apple App Store	4.6	General	n=5;<br>Allows sharing;<br>Allows password-protection; App community;<br>Geographic information;<br>Works in background;	Steps; Distance; Time;	Yes; Steps;	n=5;<br>Provide feedback on performance;<br>Prompt self-monitoring of behaviour;<br>Prompt specific goal setting;<br>Provide opportunities for social comparison;<br>Plan social support or social change;	3.40	4.50	4.00	4.00	3.98
**ActiFit – Auto Fitness Tracker (v. 2.5.3)**	MicroMovie Media GmbH<br><br>Google Play	4	General	n=5;<br>Allows sharing;<br>App community;<br>Calendarization;<br>Geographic information;<br>Works in background;	Steps; Distance; Time;	No;	n=7;<br>Provide feedback on performance;<br>Prompt self-monitoring of behaviour;<br>Prompt specific goal setting;<br>Provide opportunities for social comparison;<br>Plan social support or social change;<br>Provide contingent rewards;<br>Set graded tasks;	3.40	4.25	4.67	4.00	4.08

**Pedometer & Fitness Tracker (v. 1.8.8)**	SenseMe<br><br>Google Play	4.2	General	n=2;<br>Allows sharing;<br>Works in background;	Steps; Distance; Time;	Yes; Steps;	n=8;<br>Provide feedback on performance;<br>Prompt self-monitoring of behaviour;<br>Prompt specific goal setting;<br>Provide opportunities for social comparison;<br>Plan social support or social change;<br>Provide instruction;<br>Provided review of behavioural goals;<br>Provide information about behaviour health link;	3.00	3.25	3.00	3.83	3.27
**Livre Pedômetro-Contador Passo (v. 3.4.2)**	Free Pedometer<br><br>Google Play	4.6	General	n=3;<br>Allows sharing;<br>Has a back-office;<br>Has videos showing exercises and/or other information;	Steps; Distance;	No;	n=6;<br>Provide feedback on performance;<br>Prompt self-monitoring of behaviour;<br>Prompt specific goal setting;<br>Provide opportunities for social comparison;<br>Plan social support or social change;<br>Provide contingent rewards;	2.60	4.25	3.67	3.50	3.50
**Pedometer for Walking (v. 4.3.0)**	SimplifiedApp<br><br>Google Play	4.3	General	n=1;<br>Works in background;	Steps; Distance; Velocity;	No;	n=4;<br>Provide feedback on performance;<br>Prompt self-monitoring of behaviour;<br>Prompt specific goal setting;<br>Provide instruction;	3.20	4.25	3.33	3.67	3.61
**Pedometer 2.0 (v. 3.2.5)**	DSD<br><br>Google Play	4.1	General	n=1;<br>Works in background;	Steps; Distance; Time; Velocity;	No;	n=3;<br>Provide feedback on performance;<br>Prompt self-monitoring of behaviour;<br>Prompt specific goal setting;	3.20	4.00	3.33	3.33	3.47

**Pedometer GPS Sport (v. 2.1)**	Juan Francisco<br><br>Google Play	4.1	General	n=2;<br>Geographic information;<br>Works in background;	Distance; Time; Velocity;	No;	n=3;<br>Provide feedback on performance;<br>Prompt self-monitoring of behaviour;<br>Prompt specific goal setting;	2.80	3.75	3.00	3.33	3.22
**Pedometer - Step Counter (v. 3.1.2)**	Enjoy life more<br><br>Google Play	4.4	General	n=2;<br>Sends reminders;<br>Works in background;	Steps; Distance; Velocity;	Yes; Steps;	n=4;<br>Provide feedback on performance;<br>Prompt self-monitoring of behaviour;<br>Prompt specific goal setting;<br>Provide information about behaviour health link;	3.60	4.75	4.67	3.75	4.19
**Pedometer - Step Counter (v. 1.1.2)**	Zeopoxa<br><br>Google Play	4.1	General	n=2;<br>Allows sharing;<br>Works in background;	Steps; Distance; Time; Velocity;	No;	n=7;<br>Provide feedback on performance;<br>Prompt self-monitoring of behaviour;<br>Prompt specific goal setting;<br>Provide opportunities for social comparison;<br>Plan social support or social change;<br>Provide contingent rewards;<br>Set graded tasks;	3.60	4.75	4.67	4.33	4.34
**Pedometer & Step Counter - EasyFit Free (v. 1.3.8)**	Mario Hanna<br><br>Google Play	4.6	General	n=2;<br>Allows sharing;<br>Works in background;	Steps; Distance; Time;	No;	n=6;<br>Provide feedback on performance;<br>Prompt self-monitoring of behaviour;<br>Prompt specific goal setting;<br>Provide opportunities for social comparison;<br>Plan social support or social change;<br>Provide contingent rewards;	3.20	4.50	5.00	4.00	4.18

**Pedômetro - tanquinho treino (v. 3.6.0)**	iCare Fit Studio<br><br>Google Play	4.6	Adults (age >17 years)	n=5;<br>Allows sharing;<br>App community;<br>Has videos showing exercises and/or other information;<br>Sends reminders;<br>Works in background;	Steps; Distance; Time;	No;	n=10;<br>Provide feedback on performance;<br>Prompt self-monitoring of behaviour;<br>Prompt specific goal setting;<br>Provide opportunities for social comparison;<br>Plan social support or social change;<br>Provide contingent rewards;<br>Provide instructions;<br>Prompt practice;<br>Set graded tasks;<br>Model or demonstrate the behaviour;	3.60	4.00	5.00	3.80	4.10
**Pedómetro &Freqüência Cardíaca (v. 2.3.2)**	Guava Studio<br><br>Google Play	4.3	General	n=3;<br>Allows sharing;<br>Has videos showing exercises and/or other information;<br>Works in background;	Steps; Distance; Time;	No;	n=7;<br>Provide feedback on performance;<br>Prompt self-monitoring of behaviour;<br>Prompt specific goal setting;<br>Provide opportunities for social comparison;<br>Plan social support or social change;<br>Provide instructions;<br>Model or demonstrate the behaviour;	3.40	4.00	5.00	3.80	4.05
**Accupedo+ Pedometer (v. 2.0.6.G)**	Corusen LLC<br><br>Google Play	4.1	General	n=2;<br>Allows sharing;<br>Works in background;	Steps; Distance; Time; Velocity;	Yes; Steps;	n=5;<br>Provide feedback on performance;<br>Prompt self-monitoring of behaviour;<br>Prompt specific goal setting;<br>Provide opportunities for social comparison;<br>Plan social support or social change;	3.80	5.00	5.00	3.83	4.41

**Pedometer Calorie - Step Counter (v. 2)**	ALLTIMESOFT<br><br>Google Play	4.2	General	n=1;<br>Works in background;	Steps; Distance; Time; Velocity;	No;	n=3;<br>Provide feedback on performance;<br>Prompt self-monitoring of behaviour;<br>Provide instruction;	2.60	4.25	3.67	3.67	3.55
**Pedometer Step Counter - Fitness Tracker (v. 1.5)**	AppAspect Technologies Pvt. Ltd<br><br>Google Play	4	General	n=1;<br>Works in background;	Steps; Distance;	No;	n=3;<br>Provide feedback on performance;<br>Prompt self-monitoring of behaviour;<br>Prompt specific goal setting;	2.60	4.25	3.00	3.80	3.41
**Pedometer (v. 5.20)**	ITO Technologies. Inc.<br><br>Google Play	4.4	General	n=2;<br>Allows sharing;<br>Works in background;	Steps; Distance; Time; Velocity;	No;	n=4;<br>Provide feedback on performance;<br>Prompt self-monitoring of behaviour;<br>Provide opportunities for social comparison;<br>Plan social support or social change;	3.20	4.75	5.00	3.67	4.15
**Noom Walk Pedometer(v. 1.4.0)**	Noom Inc.<br><br>Google Play	4.1	General	n=3;<br>Allows sharing;<br>App community;<br>Works in background;	Steps;	No;	n=4;<br>Provide feedback on performance;<br>Prompt self-monitoring of behaviour;<br>Provide opportunities for social comparison;<br>Plan social support or social change;	3.20	4.25	3.67	3.33	3.61
**Accurate Pedometer (v. 4.3.0)**	Enjoy life more<br><br>Google Play	4.3	General	n=1;<br>Works in background;	Steps; Distance; Velocity;	No;	n=4;<br>Provide feedback on performance;<br>Prompt self-monitoring of behaviour;<br>Prompt specific goal setting;<br>Provide instruction;	3.20	4.25	4.00	3.67	3.78

**Simple Pedometer (Acurate) (v. 6.1)**	Hello World<br><br>Google Play	4.3	General	n=2;<br>Sends reminders;<br>Works in background;	Steps; Distance;	Yes; Steps;	n=4;<br>Provide feedback on performance;<br>Prompt self-monitoring of behaviour;<br>Prompt specific goal setting;<br>Prompt practice;	3.60	4.50	4.67	3.75	4.13
**Pedometer Step Counter (v. 5.2.2)**	SimplifiedApp<br><br>Google Play	4.5	General	n=2;<br>Sends reminders;<br>Works in background;	Steps; Distance;	Yes; Steps;	n=4;<br>Provide feedback on performance;<br>Prompt self-monitoring of behaviour;<br>Prompt specific goal setting;<br>Prompt practice;	3.60	4.50	4.67	3.25	4.00
**Step Counter - Pedometer Free & Walking Tracker (v. 1.0.19)**	Leap Fitness Group<br><br>Google Play	4.7	General	n=3;<br>Allows sharing;<br>Sends reminders;<br>Works in background;	Steps; Distance; Time;	No;	n=8;<br>Provide feedback on performance;<br>Prompt self-monitoring of behaviour;<br>Prompt specific goal setting;<br>Provide opportunities for social comparison;<br>Plan social support or social change;<br>Provide contingent rewards;<br>Prompt practice;<br>Prompt review of behavioural goals;	3.60	4.50	4.67	3.67	4.11
**Pedometer and Step Counter for Walking and Running (v. 3.0.21)**	Health & Fitness Tracker Apps<br><br>Google Play	4.1	General	n=2;<br>Allows sharing;<br>Works in background;	Steps; Distance;	No;	n=4;<br>Provide feedback on performance;<br>Prompt self-monitoring of behaviour;<br>Provide opportunities for social comparison;<br>Plan social support or social change;	3.00	4.00	3.33	4.00	3.58

**Pedometer Plus – Step Counter & Walking Tracker (v. 1.1.2)**	PVDApps<br><br>Google Play	4.1	General	n=4;<br>Allows sharing;<br>Has a back-office;<br>Requires login;<br>Works in background;	Steps; Distance; Time;	No;	n=5;<br>Provide feedback on performance;<br>Prompt self-monitoring of behaviour;<br>Prompt specific goal setting;<br>Provide opportunities for social comparison;<br>Plan social support or social change;	3.20	4.00	4.00	3.60	3.70
**Sports Tracker Running Cycling (v. 3.20.0)**	Amer Sports Digital<br><br>Google Play	4.5	General	n=7;<br>Allows sharing;<br>App community;<br>Connects with peripheral devices;<br>Geographic information; Has a back-office;<br>Requires login;<br>Works in background;	Distance; Time; Velocity;	No;	n=7;<br>Provide feedback on performance;<br>Prompt self-monitoring of behaviour;<br>Prompt specific goal setting;<br>Provide opportunities for social comparison;<br>Plan social support or social change;<br>Provide instruction;<br>Prompt review of behavioural goals;	3.60	4.00	4.67	4.00	4.07
**StepWalk Pedometer (v. 3.14)**	Fum4dev<br><br>Google Play	4	General	n=3;<br>Allows sharing;<br>Geographic information;<br>Works in background;	Steps; Distance; Time;	No;	n=4;<br>Provide feedback on performance;<br>Prompt self-monitoring of behaviour;<br>Provide opportunities for social comparison;<br>Plan social support or social change;	2.80	3.50	3.00	3.33	3.16
**Walking Odometer Pro - GPS Pedometer & Fitness (v. 1.31)**	DS Software<br><br>Google Play	4	General	n=3;<br>Allows sharing;<br>Geographic information;<br>Works in background;	Distance; Time; Velocity;	No;	n=5;<br>Provide feedback on performance;<br>Prompt self-monitoring of behaviour;<br>Prompt specific goal setting;<br>Provide opportunities for social comparison;<br>Plan social support or social change;	2.80	4.00	3.00	3.60	3.35

**WalkLogger Pedometer (v. 1.7.14)**	WalkLogger<br><br>Google Play	4.1	General	n=3;<br>Allows sharing;<br>Has a back-office;<br>Works in background;	Steps; Distance; Time;	No;	n=6;<br>Provide feedback on performance;<br>Prompt self-monitoring of behaviour;<br>Prompt specific goal setting;<br>Provide opportunities for social comparison;<br>Plan social support or social change;<br>Provide contingent rewards;	3.20	4.00	3.33	4.00	3.63

PA: physical activity; MARS: Mobile Application Rating Scale.
